# Low occurrence of *Salmonella* spp. in wild birds from a Swiss rehabilitation centre

**DOI:** 10.1002/vro2.17

**Published:** 2021-07-22

**Authors:** Barbara R. Vogler, Katrin Zurfluh, Prisca Mattmann, Kira Schmitt, Sarah Albini

**Affiliations:** ^1^ National Reference Centre for Poultry and Rabbit Diseases (NRGK) Institute for Food Safety and Hygiene Vetsuisse Faculty University of Zurich Zurich Switzerland; ^2^ Institute for Food Safety and Hygiene Vetsuisse Faculty University of Zurich Zurich Switzerland; ^3^ Swiss Ornithological Institute Sempach Switzerland

**Keywords:** salmonella schleissheim, salmonella typhimurium, wild birds, zoonoses

## Abstract

**Background:**

*Salmonella* are bacteria of the family *Enterobacteriaceae* with a wide host range. Infection in birds causes subclinical disease to mass mortality events. Wild birds may act as healthy carriers posing a hazard to livestock and humans. The present study investigated the occurrence of *Salmonella* in wild birds admitted to a rehabilitation centre in order to assess the exposure of the staff to this zoonotic pathogen.

**Methods:**

Faecal swabs of 552 avian patients (68 species) were collected over the course of 12 months. Each sample was propagated in enrichment broth and subsequently incubated on a RAPID'*Salmonella* plate. *Salmonella* isolates were serotyped, and antimicrobial susceptibility testing was performed.

**Results:**

Six *Salmonella enterica* subsp. *enterica* serovar Typhimurium (*S*. Typhimurium) and 1 *S*. Schleissheim were detected; all were pansusceptible to the antibiotics tested.

**Conclusion:**

Despite the low positive rate in the tested population, the authors recommend applying protective equipment and hygiene measures when handling wild birds.

## INTRODUCTION

*Salmonella* are Gram‐negative rod‐shaped bacteria belonging to the family *Enterobacteriaceae*.^1^ The species *Salmonella* (*S.) enterica* comprises six subspecies, which are further classified into serovars (syn. serotypes) according to their O‐ and H‐antigens (White‐Kaufmann‐Le Minor scheme).^2^ *Salmonella enterica* subsp. *enterica* mostly occurs in warm‐blooded vertebrates and currently comprises 1531 serovars.

*Salmonella* denote a wide host range in animals and humans, and almost all serovars have a zoonotic potential.^3^ However, within some serovars, there are variants/phage types with a narrow host range, such as phage types DT2 and DT99 of *Salmonella* Typhimurium, which are adapted to pigeons,^4^ and few serovars are strictly host‐adapted, such as *S*. Typhi and *S*. Paratyphi A and C in humans.^3^ Non‐host adapted (ubiquitous) *Salmonella* usually cause self‐limiting enteritis in mammals. While livestock may or may not show disease, they can become persistent shedders of *Salmonella*, thus promoting the spread to other animals or humans.^3^ Infection in humans is generally characterized by gastrointestinal symptoms accompanied by fever, myalgias, cephalalgias and malaise over 2–4 days, and recovered patients may shed *Salmonella* for up to a few months,^3^ again, aiding the spread of *Salmonella* to other individuals causing further illness and loss of revenue.

In wild birds, infection with certain *Salmonella* serovars, for example, *Salmonella* Typhimurium can lead to mass mortality events which are mainly described for passerine birds in winter. However, an infection may also take a milder clinical course or lead to clinically inapparent infection.^5^, ^6^, ^7^ Wild birds, as very mobile species, are therefore often discussed as healthy carriers of *Salmonella* spp. that could pose a hazard to livestock and humans. However, it is still unclear whether wild birds function as true reservoirs or merely act as a factor in dispersal of *Salmonellae* acquired from the environment or livestock.^6,^, ^8^, ^9^, ^10^, ^11^

Workers at wildlife rehabilitation centres present a population at risk for the transmission of zoonotic infections. Yearly between 800 and 1500 birds were admitted to the wild bird rehabilitation centre of the Swiss Ornithological Institute, Sempach, Lucerne, Switzerland during the last 10 years. Avian patients receive care (e.g., cleaning, feeding, veterinary care) depending on their condition. While manual handling is reduced to a necessary minimum, the staff of the cntre (six persons including a veterinarian) comes into close contact with the birds whenever handling is unavoidable.

The objective of the present study was to detect the *Salmonella* occurrence in wild birds admitted to the wild bird rehabilitation centre of the Swiss Ornithological Institute over the course of 1 year. The study aimed to assess the potential exposure of the workers at the rehabilitation centre to this zoonotic pathogen.

## MATERIAL AND METHODS

For the present study, faecal swabs of the avian patients at the rehabilitation centre of the Swiss Ornithological Institute were collected over the course of 12 months, between May 2018 and May 2019, namely from 552 birds representing 68 different species of 14 orders (Table S1). For all birds, an individual animal ID, the finding place, species, age and findings from the clinical assessment were noted. Age and body condition of the birds were determined by an experienced ornithologist and veterinarian. Birds were housed and cages cleaned and disinfected as detailed by Stalder et al.^12^

Swabs (Transwab Amies sterile, with Amies medium MW170; HuberLab) were taken of freshly passed faeces from a sterile surface or – in the case of dead birds – collected from the cloaca. Swabs were taken within the first 24 h after arrival of the birds at the station and prior to any treatment with antibiotics. Samples were numbered consecutively, and sample ID and sampling date were noted on the clinical record of the individual animal. Swabs were stored refrigerated for a maximum of 7 days before they were sent to the laboratory in batches.

At the laboratory, each swab was incubated in 5 ml *Enterobacteriaceae* Enrichment broth (BD, Franklin Lakes, USA) at 37°C for 24 h. One loopful of each enrichment was inoculated onto RAPID'*Salmonella* agar (RSAL; BIO RAD Laboratories, Inc., California, USA) and incubated at 37°C for 24 h. For confirmation of species identity, matrix‐assisted laser desorption/ionisation time‐of‐flight mass spectrometry (Biotyper MALDI‐TOF‐MS, Bruker Daltonics, Billerica, MA, USA) was used. *Salmonella* isolates were submitted to the National Centre for Enteropathogenic Bacteria and Listeria (NENT), Switzerland, for serotyping.^2^ Antimicrobial susceptibility for ampicillin, cefazolin, cefotaxime, amoxicillin‐clavulanic acid, cefepim, nalidixic acid, ciprofloxacin, sulfamethoxazole‐trimethoprim, fosfomycin, azythromycin, nitrofurantoin, streptomycin, kanamycin, gentamicin, chloramphenicol and tetracycline was assessed using the disk‐diffusion method according to the Clinical and Laboratory Standards Institute performance standards.^13^

## RESULTS

Samples were unevenly distributed throughout the year corresponding to the number of birds admitted to the station, with the majority of samples taken in late spring (May/June: *n* = 122–146), less samples taken in early spring and summer (March/April and July/August/September: *n* = 36–49) and the least number of samples taken in autumn and winter (October to February: *n* = 4–29 per months). Accordingly, the majority of avian patients with known age (*n* = 497) were nestlings, pulli or hatching year birds (334, 67.2 per cent). The body condition was determined in 539 birds and was mainly good (*n* = 310) or moderate (*n* = 159) and to a lesser extent poor (*n* = 70). Most birds originated from the canton Lucerne (*n* = 400/552, 73.8 per cent), and to a lesser extent from further 14 (out of 26) Swiss cantons. All birds were found north of the alps.

Six *Salmonella enterica* subsp. *enterica* serovar Typhimurium (*S*. Typhimurium) and 1 *S. enterica* subsp. *enterica* serovar Schleissheim (*S*. Schleissheim) were detected between July 2018 and April 2019, translating into a colonisation rate of 1.3 per cent (95 per cent confidence interval: 0.6–2.5 per cent). Positive samples originated from five different species, namely 3/40 (7.5 per cent, 2.2–18.7 per cent) common buzzards (*Buteo* *buteo*), 1/8 (12.5 per cent, 1.4–45.4 per cent) red kites (*Milvus* *milvus*), 1/7 (14.3 per cent, 1.6–50.1 per cent) European greenfinches (*Chloris* *chloris*), 1/49 (2.0 per cent, 0.2–9.1 per cent) house sparrows (*Passer* *domesticus*), 1/4 (25.0 per cent, 2.8–71.6 per cent) grey herons (*Ardea* *cinerea*). Six birds originated from Lucerne, and 1 common buzzard was found in the neighbouring canton of Obwalden (Table 1). All seven *Salmonella* strains were susceptible to all antibiotics tested.

**TABLE 1:** Salmonella enterica subsp. enterica serovars obtained from faecal swabs of Swiss wild birds from a rehabilitation centre

Isolates		Wild birds						
Lab‐ID	*Salmonella* Serovar	Bird Species	Common name	Collection date (dd.mm.yy)	Canton	Age	Body condition	clinical findings at submission
N18‐1351	*S*. Schleissheim	*Buteo buteo*	Common buzzard	23.07.18	OW	n.d.	good	profuse bleeding from trachea, convulsions
N18‐1433	*S*. Typhimurium	*Milvus milvus*	Red kite	27.07.18	LU	hatching year	poor	suspicion of collision trauma
N18‐2955	*S*. Typhimurium	*Chloris chloris*	European greenfinch	16.12.18	LU	n.d.	moderate	moribund
N19‐0225	*S*. Typhimurium	*Buteo buteo*	Common buzzard	28.01.19	LU	second year	poor	—
N19‐0261	*S*. Typhimurium	*Passer domesticus*	House sparrow	03.02.19	LU	n.d.	moderate	—
N19‐0410	*S*. Typhimurium	*Buteo buteo*	Common buzzard	27.02.19	LU	second year	poor	—
N19‐0950	*S*. Typhimurium	*Ardea cinerea*	Grey heron	30.04.19	LU	n.d.	good	bilateral fracture of metatarsus and tibiotarsus

Abbreviations: LU, Lucerne; n.d., not determined; OW, Obwalden.

## DISCUSSION

*Salmonella* are commonly found in the intestine of wild birds.^6^ Infection occurs via the oral route by ingestion of contaminated food or water, or by the consumption of infected prey. Infected birds may be asymptomatic, show various clinical signs and even develop fatal clinical disease.^6^ Affected birds are often emaciated,^14^ but may also die of salmonellosis in good body condition, possibly due to a short course of the disease.^6^ Commonly, salmonellosis affects adult wild birds^6^ or 1st‐year birds after their first postjuvenile body moult, but younger birds may also be affected.^14^

In this study, the body condition of birds positive for *Salmonella* was good for the common buzzard sampled in July and the grey heron, moderate for the passerine birds, and poor for the remaining three raptors. Both birds in good body condition were submitted with rather acute clinical signs not related to salmonellosis (Table 1) suggesting that these birds were carriers of *Salmonella* without being clinically affected. The three raptors with poor body condition were either found in winter (*n* =  2), when food for raptors is scarce, and many die due to lack of food or had a suspect history of collision trauma, possibly limiting the birds’ ability to catch prey. These facts suggest that at least the first two birds were again rather carriers of *Salmonella* than clinically affected. However, raptors not only act as carriers of *Salmonella*, but may also suffer from clinical salmonellosis after feeding on infected prey species. Concomitant disease is discussed to increase the susceptibility to salmonellosis.^6^ Infections with *Salmonella* have also been reported for various aquatic birds,^6,^, ^15^, ^16^, ^17^, ^18^ including an outbreak in a heron colony involving six different heron species.^19^ For both *Salmonella* positive passerine birds, a greenfinch and a house sparrow, no clinical history or age was noted. While these species are the ones reported to be the most frequently affected by passerine salmonellosis,^7^ this is often associated with multiple deaths in a small geographic area such as around bird feeders.^6,^, ^7^

In the present study, six of seven *Salmonella* strains detected were identified as *S*. Typhimurium. Apart from certain variants / phage types, such as DT2 and DT99 in pigeons,^4^ *S*. Typhimurium is non‐host‐specific as it may infect a wide array of species including humans, and it is the most common serovar reported in wild birds^6^ and responsible for passerine salmonellosis.^7^ Garden birds were suggested to be a reservoir for *S*. Typhimurium^14^ and less frequently other serotypes.

*S*. Schleissheim on the other hand, which was detected in one common buzzard in the present study, is less frequently isolated from environmental samples,^20^ mammals including humans,^21,^, ^22^ birds including a Common buzzard^23^ and reptiles^28^ (Table 2) in different countries. While animals were not reported to show clinical signs, infection in humans was associated with enteritis.^21,^, ^22^

Garden birds were suggested to be a reservoir for *S*. Typhimurium^14^ and less frequently other serotypes.

**TABLE 2:** Animal species identified to carry Salmonella Schleissheim

Class (Latin)	Species (Latin)	Species (English)	Country	Reference
Aves	*Turdus viscivorous*	mistle thrush	Sweden	^24^
	*Buteo buteo*	Common buzzard	Spain	^23^
	*Gallus gallus domesticus*	Chicken	Malaysia	^25^
	*n.d*.	Goose	Poland	^26^
Mammalia	*Homo sapiens*	Human	Turkey	^21^
	*Homo sapiens*	Human	Spain	^22^
	*Lupus canis*	Dog	Germany	^20^
	*Bos taurus*	Cattle	Poland	^27^
	*n.d*.	Game	Germany	^20^
Reptilia	*Lacerta agilis L*.	Sand lizard	Poland	^28^

Abbreviation: n.d., not described.

Wild birds are often suspected to be the source of a disease incursion into farm animals, although domestic and wild birds share the same environment, and both may get infected from similar environmental sources, or pathogen transmission may consist of spill‐over and spill‐back events among wildlife, livestock and humans.^29^ The present study investigated wild birds as possible carriers of *Salmonella* as a potential hazard to livestock and humans ‐ specifically the workers of the bird rehabilitation centre. Sampling was limited to a small geographic region, due to the fact, that wild birds were not sampled in the field, but were sampled in a resource‐effective manner after admittance to a rehabilitation centre. This centre, however, is located in the canton of Lucerne in the Swiss plateau, being the canton with the highest livestock density,^30^ and ample opportunities for interspecies contacts are provided because of widespread free‐range livestock husbandry.

Having said that, despite a non‐vaccination policy in poultry and swine, the prevalence rate of *Salmonella* spp. in Swiss livestock is low (<2% in poultry, fattening pigs) compared to other European countries.^31^, ^32^, ^33^ All isolated strains from animals are tested for antimicrobial susceptibility at the Swiss national reference laboratory, and the majority of *Salmonella* spp. isolates from cattle and poultry (2018: 62% and 83%, 2019: 76% and 78%, respectively) including all *Salmonella* Typhimurium isolates were susceptible to all antimicrobial classes tested.^33^

The detected *Salmonella* isolation rate, with two different pansusceptible *Salmonella enterica* subsp. *enterica* serotypes, was low, suggesting a low hazard for livestock and also the staff of the centre to get infected. The authors nonetheless recommend wearing protective equipment and applying a stringent hygiene management whilst handling wild birds or cleaning their cages.

## CONFLICT OF INTEREST

The authors declare that there is no conflict of interest that could be perceived as prejudicing the impartiality of the research reported.

## ETHICS STATEMENT

This study conformed to the legal requirements of Switzerland and in accordance to the guidelines of the Swiss Ornithological Institute.

## AUTHOR CONTRIBUTIONS

*Design of the study*: Sarah Albini and Barbara R. Vogler. *Sampling*: Prisca Mattmann. *Laboratory analysis*: Katrin Zurfluh and Kira Schmitt. *Data analysis*: Katrin Zurfluh, Sarah Albini and Barbara R. Vogler. *Manuscript drafting*: Barbara R. Vogler and Katrin Zurfluh. *Manuscript reviewing and editing*: Sarah Albini, Prisca Mattmann and Kira Schmitt. All authors contributed helpful comments and approved the final version of the manuscript.

## Supporting information



Supporting Information

## Data Availability

All data relevant to the study are included in the article or uploaded as supplementary information.
