# Supplementary material for: Low occurrence of Salmonella spp. in wild birds from a Swiss rehabilitation centre
Source: Vet Rec Open. 2021 Jul 22;8(1):e17. doi: 10.1002/vro2.17 (PMC8297991; doi:10.1002/vro2.17)
Supplement: Supplementary file 1 — Supporting Information [file VRO2-8-e17-s001.PDF]

Supplementary table 1: Number of individuals per bird species tested for *Salmonella*.

Order (Latin)	Family (Latin)	Species (Latin)	Species (English)	no. per species	no. per order
Accipitriformes	Accipitridae	*Accipiter nisus*	Eurasian sparrowhawk	6	
		Buteo buteo	Common buzzard	40	
		Milvus migrans	Black kite	1	
		Milvus milvus	Red kite	8	55
Anseriformes	Anatidae	*Anas platyrhynchos*	Mallard duck	34	
		Cygnus olor	Mute swan	7	
		Mergus merganser	Common merganser	6	
		Netta rufina	Red-crested pochard	1	48
Apodiformes	Apodidae	*Apus apus*	Common swift	18	
		Tachymarptis melba	Alpine swift	3	21
Charadriiformes	Laridae	*Larus michahellis*	Yellow-legged gull	4	
		Larus ridibundus	Black-headed gull	2	
	Scolopacidae	*Scolopax rusticola*	Eurasian woodcock	1	7
Ciconiiformes	Ardeidae	*Ardea cinerea*	Grey heron	4	
	Ciconiidae	*Circonia circonia*	White stork	3	7
Columbiformes	Columbidae	*Columba livia forma domestica*	Feral pigeon / Fancy pigeon	30	
		Columba palumbus	Common wood pigeon	11	
		Streptopelia decaocto	Eurasian collard dove	9	50
Coraciiformes	Alcedinidae	*Alcedo atthis*	Common kingfisher	1	1
Falconiformes	Falconidae	*Falco tinnunculus*	Common kestrel	8	
		Falco subbuteo	Eurasian hobby	1	9
Gruiformes	Rallidae	*Crex crex*	Corncrake	1	
		Fulica atra	Eurasian coot	5	
		Rallus aquaticus	Water rail	1	7
Passeriformes	Acrocephalidae	*Acrocephalus scirpaceus*	Eurasian reed warbler	1	
	Certiidae	*Certhia brachydactyla*	Short-toed tree creeper	1	

Cinclidae	*Cinclus cinclus*	White-throated dipper	1
Corvidae	*Corvus corone*	Carrion crow	30
	Corvus frugilegus	Rook	2
	Garrulus glandarius	Eurasian jay	3
	Pica pica	Common magpie	18
Emberizidae	*Emberiza citrinella*	Yellowhammer	1
Fringillidae	*Carduelis carduelis*	European goldfinch	14
	Chloris chloris	European greenfinch	7
	Carduelis citrinella	Citril finch	1
	Coccothraustes coccothraustes	Hawfinch	2
	Fringilla coelebs	Common chaffinch	9
	Fringilla montifringilla	Brambling	1
	Serinus serinus	European serin	1
Hirundinidae	*Delichon urbicum*	Common house martin	3
	Hirundo rustica	Barnswallow	6
Motacillidae	*Motacilla alba*	White wagtail	7
Muscicapidae	*Erithacus rubecula*	European robin	5
	Ficedula hypoleuca	European pied flycatcher	1
	Muscicapa striata	Spotted flycatcher	2
	Phoenicurus ochruros	Black redstart	9
Paridae	*Cyanistes caeruleus*	Eurasian blue tit	22
	Parus major	Great tit	21
	Poecile palustris	Marsh tit	1
Passeridae	*Passer domesticus*	House sparrow	49
	Passer montanus	Eurasian tree sparrow	7
Regulidae	*Regulus ignicapilla*	Common firecrest	2
Sittidae	*Sitta europaea*	Eurasian nuthatch	3
Sturnidae	*Sturnus vulgaris*	Common starling	4
Sylviidae	*Sylvia atricapilla*	Eurasian blackcap	8
	Sylvia borin	Garden warbler	1

	Turdidae	*Turdus merula*	Common blackbird	71	
		Turdus philomelos	Song thrush	4	
		Turdus pilaris	Fieldfare	4	
		Turdus viscivorus	Mistle thrush	2	324
Pelecaniformes	Ardeidae	*Bubulcus ibis*	Cattle egret	1	1
Piciformes	Picidae	*Dendrocopos major*	Great spotted woodpecker	3	
		Picus viridis	European green woodpecker	4	7
Strigiformes	Strigidae	*Asio otus*	Long-eared owl	5	
		Bubo bubo	Eurasian eagle-owl	1	
		Strix aluco	Tawny owl	3	
	Tytonidae	*Tyto alba*	Barn owl	1	10
Suliformes	Phalacrocoracidae	*Phalacrocorax carbo*	Great cormorant	1	1
				Total	548
